# Authors’ response to Ashley Roberts’ letter to the editor on aspartame and cancer

**DOI:** 10.1186/s12940-021-00789-w

**Published:** 2021-09-21

**Authors:** Philip J. Landrigan, Kurt Straif

**Affiliations:** Program for Global Public Health and the Common Good, Boston College, 140 Commonwealth Avenue / Higgins Hall, Suite 648, Chestnut Hill, MA 02467 USA

Ashley Roberts, a consultant to the Calorie Control Council, a food-industry trade group that promotes the use of aspartame and other artificial sweeteners, (https://caloriecontrol.org/about-the-council/”*)* makes several incorrect and unsubstantiated claims in his response to our recent commentary, “Aspartame and Cancer – New Evidence for Causation” [1].

Our commentary discussed the significance of a report by Tibaldi et al. (2020) who reexamined lesions from Ramazzini Institute (RI) studies of rodents exposed to aspartame [2]. Those RI studies had reported that aspartame causes dose-related increases in malignant lesions in multiple organs in rats and mice [3–5]. The studies reported additionally that prenatal aspartame exposures caused increased cancers in offspring at lower doses than in adults [4], a finding of serious concern for public health given the extensive consumption of low-calorie, aspartame-sweetened beverages by pregnant women. The goal of the study by Tibaldi et al. was to evaluate the accuracy of the RI’s diagnoses of malignancy.

Tibaldi et al.’s reevaluation included both immunohistochemical and morphological examinations of the rodent lesions. The premise underlying immunohistochemical analysis is that all cells in a malignant lesion are expected to be immunohistochemically identical - i.e., monoclonal – because they are all the direct descendants of a single transformed cell [6, 7]. By contrast, the inflammatory lymphocytes that respond to an infection are of diverse cellular origin. They are therefore not immunohistochemically identical, and instead are polyclonal. Tibaldi et al’s immunohistochemical analysis employed a battery of nine markers: Ki67, CD3, PAX5, CD20, CD68, TdT, CD45, CD14 and CD33.

Tibaldi et al confirmed that 72 of the 78 lesions originally diagnosed by RI as malignant were, in fact, monoclonal. Tibaldi et al. thus confirmed the original RI diagnoses of malignancy in 92.3% of the aspartame-exposed rodents [2].

Roberts’ critique is based on his review of the histological slides presented by Tibaldi et al. and on the unsubstantiated and previously refuted [8, 9] claim that there was infection in the RI laboratories that caused inflammatory lesions in the experimental animals. He ignores Tibaldi et al.’s immunohistochemical findings and does not attempt to rebut them.

Roberts also fails to acknowledge, rebut or offer any alternative explanation for the dose-response relationship between aspartame dose and cancer incidence that was clearly evident in the RI studies and was confirmed by Tibaldi et al. Increasing aspartame exposures were associated with statistically significant increases in incidence of all hematolymphatic malignancies (*p* = 0.006), including both lymphomas (*p* = 0.032) and leukemias (*p* = 0.031) [2]. Infection would seem an unlikely explanation for such a trend.

Likewise, Roberts fails to acknowledge, rebut or explain away the RI finding that prenatal exposures to aspartame caused increased malignancies in offspring at lower doses than in adults [4]. Again, Infection would seem an unlikely explanation for this finding.

In summary, we find Robert’s critique to be flawed and unconvincing. We therefore reiterate our conclusion that national and international public health agencies need to follow the example of the International Agency for Research on Cancer (IARC) [9] and take careful notice of these new data on the carcinogenicity of aspartame [2]. These agencies need to pay particular heed to the potential of lower-dose aspartame exposures during pregnancy to cause increased incidence of leukemia and lymphoma in offspring [4].

Facile dismissals of the carcinogenicity of aspartame put forth by vested interests can no longer be sustained. Such unsubstantiated claims can impede public health interventions and can lead to unnecessary cancers, including childhood cancers and premature deaths.

## Data Availability

Not applicable.
